# SG-APSIC1058: Microbiological surveillance of endoscopes in a Singapore tertiary-care academic hospital: A retrospective study from 2018 to 2021

**DOI:** 10.1017/ash.2023.94

**Published:** 2023-03-16

**Authors:** Zhimin Zhang, Molly How Kue Bien, Lee Lai Chee, Nenny Suzanah Binte Sellamat, Chua Puay Hoon, Lai Kai Mun, Ling Moi Lin

**Affiliations:** Singapore General Hospital, Singapore; Singapore General Hospital, Singapore; Singapore General Hospital, Singapore; Singapore General Hospital, Singapore; Singapore General Hospital, Singapore; Singapore General Hospital, Singapore; Singapore General Hospital, Singapore

## Abstract

**Objectives:** Improper reprocessing of endoscopes may result in healthcare-associated infections. Regular microbiological surveillance is an important means of evaluating the quality of endoscope reprocessing. We evaluated the effectiveness of reprocessing endoscopes (including the protocols on steps to be taken in the event of any positive microbiological results) in a sterile supply unit (SSU) and an endoscopy unit in a Singapore tertiary-care academic hospital. **Methods:** Singapore General Hospital (SGH) is a 1,750-bed, tertiary-care, academic medical center in Singapore with 2 main SSUs: 1 inpatient endoscopy unit and 1 outpatient endoscopy unit. We reviewed microbiological surveillance results from endoscopes following reprocessing from January 2018 to December 2021. In total, 160 endoscopes (27 bronchoscopes, 58 gastroscopes, 52 colonoscopes, 6 duodenoscopes, 5 echoscopes, 5 cystoscopes, 5 rhinolaryngoscopes, and 5 enteroscopes) and 15 automated endoscope reprocessors (AERs) were evaluated for the presence of microorganisms. Samples were obtained by swabbing the tip of the scope and the biopsy channel. Fluid was flushed from the biopsy channel after reprocessing, and this water from the AERs was sampled after waterline disinfection. **Results:** Of the 15,783 samples collected, 15,667 (99.3%) yielded no growth; 36 (0.2%) were positive for gut and environmental flora; and 80 (0.5%) were positive for low-concern organisms such as skin flora. **Conclusions:** Microbiological surveillance yielded a high percentage of negative results confirming the effectiveness of endoscope reprocessing. This quality-assurance process is necessary and beneficial in achieving patient safety.

